# Impact of Alkyl Side Chain Length on Morphological Properties and Magnetic Field Response Characteristics of Naphthalenediimide-Based Conjugated Polymer

**DOI:** 10.3390/polym18111328

**Published:** 2026-05-27

**Authors:** Shichao Chen, Yiqian Zhou, Zitong Zhang, Xiaocan Zhang, Di Hui, Yuhan Zhang, Yurou He, Kai Zhang, Yingzheng Ge, Ziyan Feng, Lin Hu, Chun Ye, Guoxing Pan

**Affiliations:** 1 Institutes of Physical Science and Information Technology, Anhui University, Hefei 230601, China; chenshichao2023@163.com (S.C.); zhouyiqian0728@163.com (Y.Z.); ztzhang2024@163.com (Z.Z.); xczhang20000527@163.com (X.Z.); zhangyuhanahu@163.com (Y.Z.); heyurouahu@163.com (Y.H.); geyz0202@163.com (Y.G.); fzy20250919@163.com (Z.F.); 2 Anhui Province Key Laboratory of Condensed Matter Physics at Extreme Conditions, High Magnetic Field Laboratory (HMFL), Chinese Academy of Sciences, Hefei 230031, China; huidi021@163.com (D.H.); zeki@mail.ustc.edu.cn (K.Z.); hulin@hmfl.ac.cn (L.H.)

**Keywords:** alkyl side chain length, high magnetic field, alignment, aggregation, OFET

## Abstract

The molecular structure and magnetic properties of two conjugated polymer molecules, which have the same core of naphthalene diimide (NDI) but varying alkyl side chain lengths of 2-hexyldecyl (P(NDI2HD-T2)) and 2-octyldodecyl (P(NDI2OD-T2)), are compared. Microstructural characterizations revealed that the P(NDI2HD-T2) film exhibits a shorter π-π stacking distance and more pronounced crystalline behaviors when compared to the P(NDI2OD-T2) film. In addition, the magnetically aligned P(NDI2HD-T2) film exhibited a higher degree of chain alignment compared to the P(NDI2OD-T2) film grown using the same film preparation method. The organic field-effect transistor (OFET) based on the resulting P(NDI2HD-T2) film exhibited an average electron mobility of 1.49 cm^2^ V^−1^ s^−1^, which is about a 13.5-fold enhancement compared to the spin-coat film. The findings of our study offer valuable insights into the process of magnetic manipulation, thereby offering guidelines for the rational selection of polymers to fabricate highly ordered films via the magnetic alignment method.

## 1. Introduction

Solution-processable semiconducting polymers have received great attention in flexible electronic devices, including integrated circuits [1,2], flexible displays [3], memory devices [4], and sensors [5,6]. The carrier mobility of polymers has witnessed remarkable advancements spanning several orders of magnitude in recent years, owing to the rational design of molecules and the adoption of more suitable processing techniques. Generally, the carrier transport is believed to be most efficient along the backbone chains [7,8,9]. Thus, the unidirectional alignment of the polymer chains is considered to be effective at enhancing the field-effect mobility, and various methods for aligning semiconducting polymers have been reported, such as blade coating [10,11], dip coating [12], off-center coating [13], high-temperature rubbing [14], brush printing [15], bar coating [16], and others. However, the majority of the films produced using these alignment techniques exhibit a limited extent of polymer film alignment and uniformity across large areas.

Recently, the magnetic field has demonstrated considerable potential for manipulating molecular orientation, which enables the alignment of polymer chains over large areas to improve carrier transport. The driving force for magnetically aligned semiconducting polymers is generated by the sufficiently anisotropic diamagnetic susceptibility [17,18,19,20]. Our research group successfully achieved highly aligned and uniform films of D-A semiconducting polymers via a magnetic alignment method, exemplified by P(NDI2OD-T2) [20,21,22] and PDVT-8 [23]. These research findings have convincingly demonstrated the efficacy of the magnetic alignment method in fabricating highly aligned polymer films. Up to now, we have held the opinion that the pre-aggregation behavior of donor–accepter (D-A) polymers in solution plays an important role in the magnetic alignment [20,21,22,23]; however, the movement of polymer molecules in a magnetic field is a complex phenomenon, and the precise alignment process and underlying mechanisms remain incompletely understood. Thus, it is imperative to gain a deeper understanding of the mechanism underlying magnetic field–induced molecular orientation self-assembly and fabricate polymer films with a higher degree of orientation structure.

It has been demonstrated that preaggregates in solution not only serve as the fundamental controlling unit during the magnetic alignment process but also exert a significant influence on the microstructure of the solid film through their memory effect [24,25]. So, it is of paramount importance to regulate the pre-aggregation behavior of polymer molecules in solution during the magnetic alignment process. In fact, the pre-aggregation behavior of D-A copolymers is indeed influenced by various parameters, including the side chain structure [26,27,28], solvent [29], temperature [23], and molecular weight [23,30]. Among various factors, alkyl side chain length plays a crucial role in determining the properties of D-A copolymer films [31,32,33]. This is because the alkyl side chains not only function as solubilizing groups that modify molecular solubility but also influence interchain π-interactions, pre-aggregation behavior and molecular packing [34]. Significantly, extensive research has been conducted on the influence of alkyl side chain length on pre-aggregation and electrical properties in D-A copolymers. However, limited attention has been given to comprehending the variation in magnetic responsiveness of polymers with varying alkyl side chain lengths.

Herein, we investigate the impact of alkyl side chain length on pre-aggregation behavior, molecular packing, electrical performance, and magnetic responsiveness by examining two homologous NDI-based polymers with different alkyl side chain lengths of 2-hexyldecyl ((poly{[N,N′-bis(2-hexyldecyl)-naphthalene-1,4,5,8-bis(dicarboximide)-2,6-diyl]-alt-5,5′-(2,2′-bithiophene)}, P(NDI2HD-T2)) and 2-octyldodecyl (poly{[N,N′-bis(2-octyldodecyl)-naphthalene-1,4,5,8-bis (dicarboximide)-2,6-diyl]-alt -5,5′-(2,2′-bithiophene)}, P(NDI2OD-T2)). The two-dimensional grazing incidence wide-angle X-ray scattering (2D-GIWAXS) results clearly demonstrate that P(NDI2HD-T2) exhibits a significantly shorter π-π stacking distance and stronger crystalline behaviors compared to P(NDI2OD-T2). Additionally, P(NDI2HD-T2) demonstrated a higher magnetic field responsiveness in comparison to P(NDI2OD-T2). Thus, the magnetically aligned P(NDI2HD-T2) films exhibit an elevated degree of molecular orientation, with a dichroic ratio reaching as high as 25.2. The corresponding OFETs fabricated from the aligned P(NDI2HD-T2) films displayed a remarkably high mobility of 1.49 cm^2^ V^−1^ s^−1^. The results obtained using the NDI-based system model can provide valuable insights for the design of other donor–accepter polymers, aiming to enhance the responsiveness of polymer molecules toward a magnetic field.

## 2. Materials and Methods

### 2.1. Materials and Sample Preparation

Both the P(NDI2ODT2) and P(NDI2HD-T2) were purchased from 1-Material Inc. (Dorval, QC, Canada) and used as received without further purification. The solutions (8 mg/mL) were prepared by dissolving the conjugated polymers in dichlorobenzene (DCB). The substrates (Si/SiO_2_ and quartz) were cleaned subsequently by ultrasonification in acetone and isopropanol, followed by oxygen plasma treatment. The solutions were spin-coated on Si/SiO_2_ and quartz substrates at a speed of 1000 rpm for 60 s. Then, the as-cast wet films were quickly placed vertically in a sealed glass vial filled with DCB vapor, and solvent vapor annealing was performed under a vertical magnetic field of 10 T at room temperature for 6 h, similar to our previously reported research [21,22,23]. All films were annealed at 140 °C under a N_2_ atmosphere for 1 h.

### 2.2. Characterizations

A MAPADA M9 spectrophotometer was used to measure ultraviolet-visible (UV-vis) absorption. The molecular weight of polymer distributions was estimated by gel-permeation chromatography (GPC) on Agilent 1260lnfinity II HT GPC (Santa Clara, CA, USA). The 2D-GIWAXS measurements were carried out at the Shanghai Synchrotron Radiation Facility (SSRF, Shanghai, China) on beamline BL14B. The X-ray energy was set to 10 keV, corresponding to a wavelength of λ = 1.24 Å, and the incidence angle was fixed at 0.2°. The instrument is equipped with a two-dimensional MAR CCD detector. The magnetic properties were measured using a superconducting quantum interference device (SQUID)/Magnetic Property Measurement System (MPMS3, Quantum Design, San Diego, CA, USA). The morphology of the prepared films was examined using a HITACHI-5500M atomic force microscope (AFM) in tapping mode.

### 2.3. Fabrication and Characterization of OFET Devices

The top-gate/bottom-contact (TG/BC) OFET devices were fabricated on Si/SiO_2_ substrates. The substrates were subjected to photolithography to deposit a 30 nm thick Au layer, forming source/drain electrode arrays with a channel width of 2 mm and a channel length of 5 μm. The aligned films were prepared as described above. Thereafter, a CYTOP (CTL-809M, AGC Corporation, Tokyo, Japan) insulator was spin-coated on the films as the dielectric layer, followed by annealing at 100 °C for 1 h. Finally, Ag (80 nm) was evaporated as the gate electrode. The electrical performance of OFETs was measured in a N_2_-atmosphere glove box using a Keithley 2612A. The mobility was calculated from the saturation region using the following equation:

$$I_{D}=\frac{WC_{i}\mu (V_{G}-V_{T}{)}^{2}}{2L}$$

where W and L are the channel width and length, respectively, Ci is the area capacitance of the CYTOP dielectric (2.85 nF/cm^2^), V_G_ and V_T_ are the gate and threshold voltages, respectively, and µ is the charge carrier mobility.

## 3. Results and Discussion

### 3.1. Morphologies of Magnetically Aligned Polymer Films

**Figure 1:**
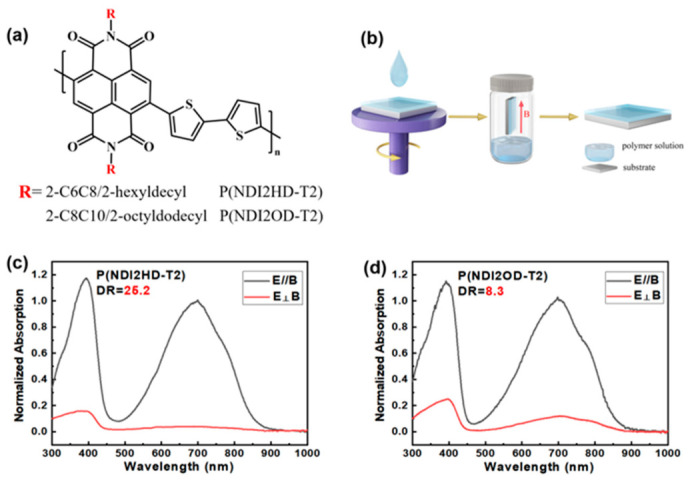
(**a**) The diagrams of molecular structures for P(NDI2HD-T2) and P(NDI2OD-T2); (**b**) the schematic of the SVA-HMF process, the red arrow denote the magnetic field direction; polarized UV-vis absorption spectra of the magnetically aligned P(NDI2HD-T2) (**c**) and P(NDI2OD-T2) (**d**) films. “E//B” and “E⊥B”, referred to as the incident light, are parallel and perpendicular to the applied HMF direction, respectively.

The molecular structure of the NDI-based polymer with two different alkyl side chains utilized in this study is depicted in Figure 1a. The number-average molecular weight (Mn) and polydispersity index (PDI) were determined to be 25.1 kDa (PDI = 2.3) for P(NDI2HD-T2) and 24.3 kDa (PDI = 1.8) for P(NDI2OD-T2), respectively (Figure S1, Supporting Information). Both types of polymers exhibited comparable Mn and PDI values, thereby mitigating the impact of Mn on the experimental results. The P(NDI2HD-T2) and P(NDI2OD-T2) were magnetically aligned through a solvent vapor annealing process in DCB vapor under a high magnetic field of 10 T (SVA-HMF), as depicted in Figure 1b. The thickness of magnetically aligned films was measured to be within the range of 45–55 nm, as shown in Figure S2. To clarify whether the SVA-HMF films of P(NDI2HD-T2) and P(NDI2OD-T2) were oriented, the surface was measured by polarized optical microscopy (POM), as shown in Figure S3. The POM images of both films exhibit a transition from brightness to darkness as the axis of the applied magnetic field is rotated from 45° to 0° relative to the polarizer axis. The presence of birefringence unequivocally indicates that the polymer chains in the films exhibit a highly aligned orientation.

To quantify the alignment degree of SVA-HMF NDI-based films, we additionally employed polarized UV-vis absorption spectroscopy. The alignment degree of the polymer backbone can be obtained by the dichroic ratio DR = A_//_/A_⊥_, where A_∥_ and A_⊥_ represent the absorbance parallel and perpendicular to the direction of HMF, respectively [35]. The polarized absorbance spectra of SVA-HMF P(NDI2HD-T2) and P(NDI2HD-T2) films, as shown in Figure 1c,d, both exhibit a pronounced absorption within the range of 500–900 nm with an absorption peak centered around 700 nm. The DR of the SVA-HMF P(NDI2OD-T2) film is found to be as high as 8.3. Because the transition dipole is approximately along the polymer chain backbone [36], the high DR value serves as evidence for a highly aligned orientation of the P(NDI2OD-T2) chains in the HMF direction. The DR of SVA-HMF P(NDI2OD-T2) film obtained in this study is higher than that reported in our previous work (DR = 5.9), primarily due to the utilization of a stronger magnetic field (10 T vs 9 T). Regarding the SVA-HMF P(NDI2HD-T2) film, its DR reaches an impressive value of 25.2, surpassing more than three times that of the SVA-HMF P(NDI2OD-T2) film. In the case of the same film preparation process, the significant disparity in the degree of orientation between the two films suggests that reducing the alkyl side chain length of NDI-based polymers can considerably enhance the molecular responsiveness to the magnetic field, thereby achieving a substantial improvement in polymer film orientation.

**Figure 2:**
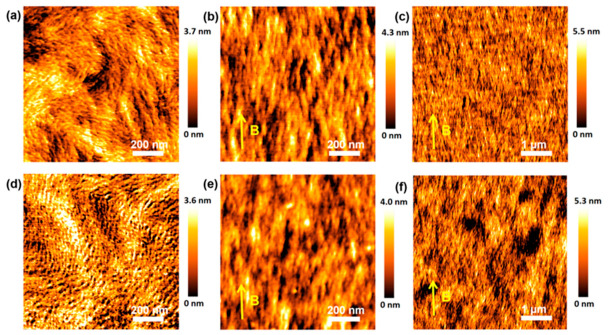
AFM height images of P(NDI2HD-T2) (**a**–**c**) and P(NDI2OD-T2) (**d**–**f**) films. The films were prepared by the spin-coat (**a**,**d**) and SVA-HMF (**b**,**c**,**e**,**f**) methods, respectively. The yellow arrows denote the HMF direction.

Furthermore, the atomic force microscopy (AFM) measurements were employed for the characterization of alterations in both morphology and molecular orientation of polymer films induced by variations in alkyl side chain length. The AFM height and phase images of spin-coat P(NDI2HD-T2) and P(NDI2OD-T2) films are presented in Figure 2 and Figure S4, respectively, revealing a randomly distributed fibrillar microstructure consistent with previous studies. Additionally, it is observed that the fibrils in P(NDI2HD-T2) film are larger than those of the P(NDI2OD-T2) film, suggesting that shortening the alkyl side chain length of the polymer molecule can improve polymer crystallization and enhance fibril size. And as shown in Figure 2b,e, the SVA-HMF P(NDI2HD-T2) and P(NDI2OD-T2) films both exhibit highly oriented fiber microstructures that are uniformly aligned along the direction of the magnetic field, accompanied by an increase in fiber size relative to their respective spin-coat films. The reason for this phenomenon lies in the fact that, during the SVA-HMF process of film formation, the highly oriented preaggregates undergo further aggregation, resulting in an enlargement of the fiber size within the solid film. It is worth noting that while both the SVA-HMF P(NDI2HD-T2) and P(NDI2OD-T2) films exhibit a similar high degree of orientation structure within a small range (1 µm × 1 µm), the orientation degree of the SVA-HMF P(NDI2HD-T2) film surpasses that of the SVA-HMF P(NDI2OD-T2) film significantly across a larger range of AFM images (5 µm × 5 µm), as illustrated in Figure 2c,f and Figure S4c,f. The microstructures of the SVA-HMF films are in accordance with the findings from UV-vis analyses. Based on these results, it can be inferred that the microstructure of the films prepared using the SVA-HMF method is significantly influenced by the alkyl side chain length of the polymer molecules.

### 3.2. Crystallinity Analysis of Polymer Films

**Figure 3:**
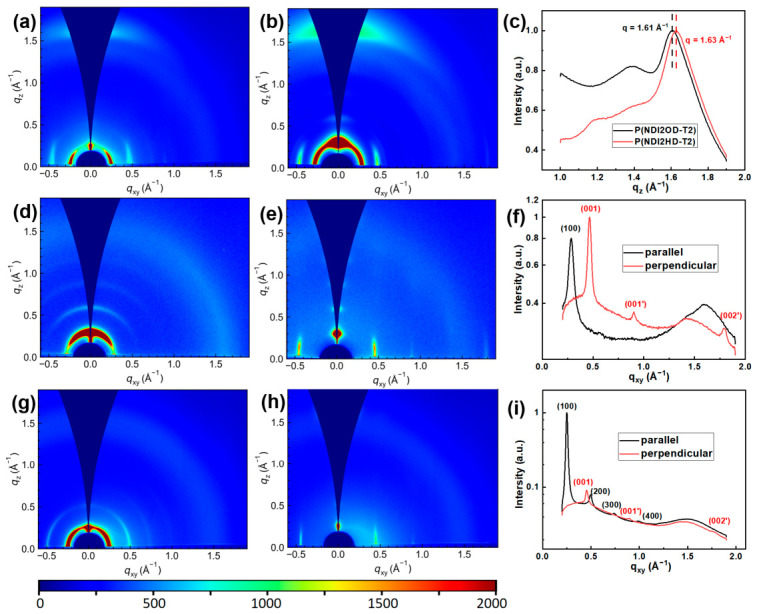
The 2D-GIWAXS images of the P(NDI2OD-T2) (**a**,**g**,**h**) and P(NDI2HD-T2) (**b**,**d**,**e**) films grown via spin-coating (**a**,**b**) and SVA-HMF (**d**,**e**,**g**,**h**). The X-ray beam is parallel (**d**,**g**) and perpendicular (**e**,**h**) to the HMF direction. (**c**) Cross-section profiles along the out-of-plane orientation of the 2D-GIWAXS patterns shown in (**a**,**b**); (**f**,**i**) are the cross-section profiles along the in-plane orientation of the (**d**,**e**) and (**g**,**h**), respectively.

The crystalline nature and molecular packing of the NDI-based polymers with different alkyl side chains were studied by 2D-GIWAXS measurements, as shown in Figure 3. First, the 2D-GIWAXS patterns of the P(NDI2HD-T2) exhibit more scattering signals with higher intensities compared to P(NDI2OD-T2). Therefore, it can be concluded that thin films of P(NDI2HD-T2) show better crystallinities than those of P(NDI2OD-T2). In addition, in the 2D-GIWAXS patterns of the spin-coat P(NDI2HD-T2) and P(NDI2OD-T2) films (Figure 3a,b), the (h00) reflections were prominently observed in both the out-of-plane and in-plane direction, indicating a mixed texture with coexisting face-on and edge-on crystallites [37,38,39]. As the alkyl side chain length decreases, the polymers exhibit a decreasing trend in the lamellar spacings (d100) of 25.1 (q_xy_ = 0.25 Å^−1^) and 22 Å (q_xy_ = 0.285 Å^−1^) for P(NDI2OD-T2) and P(NDI2HD-T2), respectively. Furthermore, both P(NDI2HD-T2) and P(NDI2OD-T2) showed (001) and (00l’) scattering signals in the in-plane direction (Figure 3a,b), suggesting a typical kinetically trapped form I stacking mode for P(NDI2HD-T2) and P(NDI2OD-T2) molecules; that is, the NDI units of one molecule tend to stack on the NDI units of the other molecule [39,40,41]. In addition, a reduction in the π-π stacking distances was observed from 3.9 Å (qz = 1.61 Å-1, P(NDI2OD-T2)) to 3.85 Å (qz = 1.63 Å-1, P(NDI2HD-T2)) as the alkyl side chain length decreased. The shorter π-π stacking distance observed in P(NDI2HD-T2) can be attributed to its more pronounced planar structure and enhanced intermolecular interactions. And the crystalline correlation length of the backbone was calculated from the (001) scattering signal (CCL_001_) using the Scherrer formula (Figure S5). The CCL_001_ values showed remarkable increases from 6.34 (P(NDI2OD-T2)) to 10.5 nm (P(NDI2HD-T2)). These results unequivocally demonstrate that reducing the alkyl side chain length of NDI-based polymers significantly enhances the film crystallinity.

Additionally, for the anisotropic films of SVA-HMF P(NDI2OD-T2) and P(NDI2HD-T2), the 2D-GIWAXS patterns show significant differences in both the position and intensity of scattering signals when the incident X-ray beam is parallel or perpendicular to the direction of the applied magnetic field. And the differences are particularly evident in the cross-sectional patterns shown in Figure 3f,i. Specifically, for the perpendicular direction of SVA-HMF P(NDI2OD-T2) and P(NDI2HD-T2) films, only the (00l) series reflections of backbone repeats exist, and the (00l) peaks disappear completely in the case of the parallel direction. What is more obvious is that when the parallel changes to the perpendicular, the (100) peaks in the out-of-plane direction change from a very wide arc to a point. Based on the distinct signal characteristics observed in the parallel and perpendicular directions of the SVA-HMF P(NDI2OD-T2) and P(NDI2HD-T2) films, it was indicated that highly anisotropic NDI-based polymer films have been prepared by a magnetically aligned method, and the direction of the polymer backbones was highly parallel to the direction of the applied high magnetic field. Furthermore, the scattering signals from the SVA-HMF films demonstrate significantly higher intensity and notably sharper profiles in comparison to those of the spin-coated films, and the CCL_001_ of the SVA-HMF P(NDI2OD-T2) and P(NDI2HD-T2) films have expanded to 12.6 nm and 15.8 nm, respectively (Figure S5). In order to further quantify the differences in the degree of crystallinity among series films, the relative degree of crystallinity (rDoC) needs to be obtained [42,43]. The rDoC can be characterized in terms of the S_SVA-HMF_/S_spin-coated_ ratio, where SSVA-HMF and Sspin-coated denote the peak area of (001) reflection for the SVA-HMF and spin-coated films, respectively. The ratio values of P(NDI2OD-T2) and P(NDI2HD-T2) are 2.5 and 4.2, respectively. The fact that both values are larger than 1 indicates that the SVA-HMF process can enhance the degree of order and crystallinity in the film. Additionally, the higher rDoC of P(NDI2HD-T2) shows that reducing the alkyl side chain length of NDI-based polymers can considerably enhance the molecular responsiveness to the magnetic field, thereby achieving a more significant increase in the polymer film degree of crystallinity. This result is consistent with the polarized UV-vis absorption spectroscopy and AFM measurements.

### 3.3. Density Functional Theory Calculations

**Figure 4:**
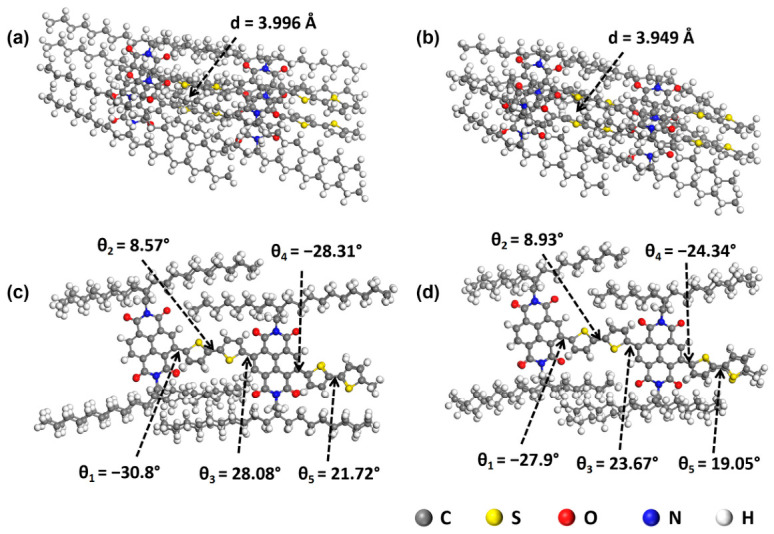
The optimized geometries of P(NDI2OD-T2) (**a**,**c**) and P(NDI2HD-T2) (**b**,**d**) are presented in the side view (**a**,**b**) and top view (**c**,**d**).

To elucidate the molecular mechanisms underlying the dependence of molecular packing on alkyl side chain length, we performed a comparative analysis of the molecular packing properties of P(NDI2HD-T2) and P(NDI2OD-T2) using density functional theory (DFT) calculations. The DFT calculations were carried out with the Vienna ab initio Simulation Package (VASP) code [44]. The Perdew–Burke–Ernzerhof (PBE) functional within generalized gradient approximation (GGA) [45,46] was used to process the exchange–correlation functional. We set the kinetic energy cut-off at 500 eV; the structure relaxation process was continued until the convergence criteria of 10^–5^ eV for energy and 0.05 eV Å-1 for force were achieved, respectively. A vacuum layer with a thickness of 15 Å was introduced to eliminate any potential interactions between the periodic images in the surface models. Grimme’s DFT-D3 method was employed to correct the van der Waals (vdW) interactions [47]. We performed structure optimizations and compared the structural features of P(NDI2HD-T2) and P(NDI2OD-T2), including d-spacing and torsion angle. The optimized structures of the two materials are shown in Figure 4. The d-spacing values of P(NDI2OD-T2) and P(NDI2HD-T2) were 3.996 and 3.949 Å, respectively (Figure 4a, Figure 4b). The findings suggest that P(NDI2HD-T2) exhibits a stacking structure with a shorter π-π packing distance relative to P(NDI2OD-T2), which is consistent with the 2D-GIWAXS results. Further, we measured the torsional angles between the NDI ring and the thiophene in P(NDI2DT-T2) and P(NDI2HD-T2) to compare the coplanarity of the two materials. The torsional angles in P(NDI2HD-T2) are smaller than those in P(NDI2DT-T2) (Figure 4c,d), indicating that P(NDI2HD-T2) exhibits a higher degree of molecular backbone coplanarity compared to P(NDI2DD-T2). Based on the findings from the DFT calculations, it can be concluded that reducing the alkyl side chain length effectively decreases π-π stacking distances and enhances the coplanarity of polymer molecules.

### 3.4. Impact of Alkyl Side Chain Length on Magnetic Alignment

**Figure 5:**
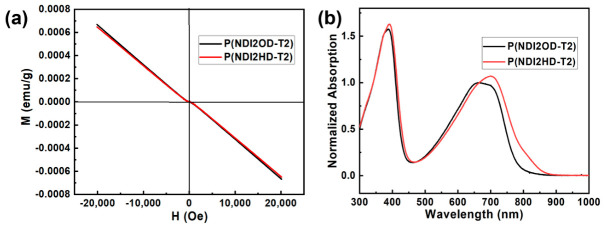
(**a**) Magnetization curves of P(NDI2HD-T2) and P(NDI2OD-T2). (**b**) UV-vis absorption spectra of P(NDI2OD-T2) (black line) and P(NDI2HD-T2) (red line) in dilute DCB (concentration: 0.02 mg mL^−1^).

As is widely acknowledged, magnetic alignment can be achieved when the magnetic energy (VB2/2*μ_o_*) significantly exceeds the thermal energy (kBT). Here, μ_0_ represents the magnetic permeability of vacuum, V denotes the volume of the molecular aggregate, B is the magnetic field strength, Δχ is the magnetic susceptibility, kB denotes the Boltzmann constant, and T stands for the temperature [17]. According to the aforementioned equation, the response of polymer molecules to magnetic fields appears to be contingent solely on their diamagnetic susceptibility and the degree of pre-aggregation. First, SQUID measurements were conducted to gain insights into the magnetic properties of the two NDI-based polymers. As illustrated in Figure 5a, the magnetization curves of P(NDI2OD-T2) and P(NDI2HD-T2) exhibit diamagnetic susceptibilities of 3.34 × 10^−8^ and 3.25 × 10^−8^ emu g^−1^ Oe^−1^, respectively. These nearly identical diamagnetic susceptibilities indicate that alkyl side chain length has a negligible effect on the magnetic properties of the NDI-based polymers. Next, we investigated the impact of alkyl side chain length on the pre-aggregation behaviors of NDI-based polymers in solution through UV-vis absorption spectroscopy. As shown in Figure 5b, both high- and low-energy bands ascribed to π-π* transition (nearly 390 nm) and intramolecular charge transfer transition (500–900 nm), respectively, were observed in the two samples. As illustrated in Figure S6, the optical bandgap of P(NDI2OD-T2) and P(NDI2HD-T2) is 1.46 eV and 1.45 eV, respectively. And P(NDI2OD-T2) exhibited a peak max at 657 nm of the charge transfer transition band with a low energy shoulder peak appearing at around 702 nm, characteristic of aggregate absorption [48,49]. However, compared to P(NDI2OD-T2), the absorption maxima of P(NDI2HD-T2) in solution are slightly red-shifted by 8 nm (665 nm), accompanied by an enhancement of the shoulder peak at 703 nm. Such differences in UV-vis absorption spectra indicated that the NDI-based polymer chains obtained a higher degree of pre-aggregation within solutions, induced by a decrease in alkyl side chain length [50]. In addition, the red shift in the absorption peak assigned to the typical ICT state also stems from the more planarized chain conformation. To be more specific, shortening the length of the side chain reduces the steric hindrance effect. This reduction not only results in a decrease in the π-π stacking distance but also causes a slight twist in the backbone chains, which is manifested as a reduction in the torsional angles between the NDI units and the thiophene unit within the polymer molecules [51]. The increase in the planarity of polymer molecules enhances their conjugation degree and ultimately results in a red shift in the absorption spectrum. In fact, for the majority of D-A semiconductor polymers featuring rigid coplanar π-conjugated structures, according to the Hartree–Fock theory for closed-shell systems, it is well established that π-conjugated structures display diamagnetism [52,53]. The axis of maximum diamagnetic susceptibility is approximately along the normal direction of the conjugated plane. Therefore, when the main chain of D-A semiconductor polymers only needs to be parallel to the direction of the magnetic field, the system can reach the minimum potential energy. 2D-GIWAXS and UV-vis absorption spectra analysis have revealed that shortening the alkyl side chain length leads to a decrease in the π-π stacking distance between adjacent molecules, enhances the degree of chain pre-aggregation, and increases the size of aggregates in solution. The consideration of the response of polymer molecules to magnetic fields mainly depends on the degree of pre-aggregation. This change in pre-aggregation enables the polymer molecules with shorter alkyl side chains in the SVA-HMF process to exhibit more pronounced magnetic anisotropy and higher anisotropic magnetic energy (greater than k_B_T), thereby enhancing their responsiveness to HMF and triggering magnetic alignment. In a word, the alkyl side chain predominantly influences the polymers’ pre-aggregate property and molecular π-π stacking distance, thereby modifying the response characteristics of polymer molecules to magnetic fields.

### 3.5. OFET Device Characterization

**Figure 6:**
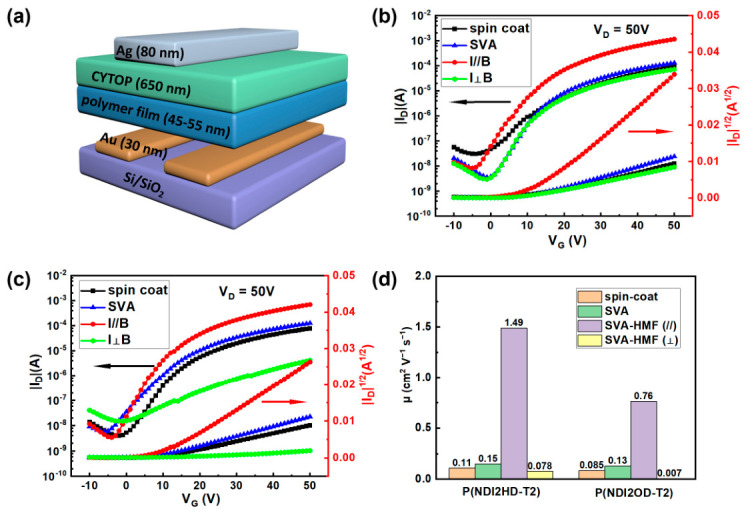
(**a**) Schematic configuration of the top-gate/bottom-contact (TG/BC) OFET device. The transfer curves of P(NDI2HD-T2) (**b**) and P(NDI2OD-T2) (**c**) OFETs based on the spin-coat, SVA and SVA-HMF films. The I//B and I⊥B indicate the channel current flow parallel and perpendicular to the HMF direction (B), respectively. (**d**) The electron mobility of the NDI-based films under different conditions.

To study the electrical characteristics of NDI-based polymer films with different alkyl side chain lengths and deposition methods, TG/BC OFETs (Figure 6a) with NDI-based polymer films as the semiconductor layer were fabricated. The transfer curves of various OFETs are shown in Figure 6, and the corresponding output curves can be found in Figures S7 and S8. The mobility values provided were extracted from the saturation regime and are summarized in Table 1. All the NDI-based polymer film devices show typical N-type characteristics. As evidenced in Table 1 and Figure 6, the electron mobilities of spin-coat P(NDI2HD-T2) and P(NDI2OD-T2) are 0.11 and 0.085 cm^2^ V^−1^ s^−1^, respectively, demonstrating that the decrease in alkyl side chain length leads to a systematic increase in OFET mobility. Therefore, reducing the alkyl chain length from 2-octyldodecyl to 2-hexyldecyl increases the charge carrier mobility; this is mainly because the shorter alkyl side chains reduce the steric hindrance between polymer chains, thus resulting in a shorter π-π stacking distance and an enhanced degree of film crystallization. As anticipated, the SVA process can enhance mobilities in these two polymers by promoting an increased degree of crystallization within the film; the average electron mobilities can reach 0.13 cm^2^ V^−1^ s^−1^ and 0.15 cm^2^ V^−1^ s^−1^ for films of P(NDI2OD-T2) and P(NDI2HD-T2) fabricated by SVA for 6 h without the HMF.

As for the magnetic alignment films, the parallel electron mobility (μ_//_) of the SVA-HMF P(NDI2OD-T2) films was up to 1.49 cm^2^ V^−1^ s^−1^, a 13.5-times improvement compared with that for the isotropic OFET of the spin-coat film. Comparatively, the mobility values of μ_//_ = 0.76 cm^2^ V^−1^ s^−1^ are measured for transistors made from SVA-HMF P(NDI2OD-T2) film, and the μ_//_/μ_spin_ value is 8.9. The significant electron mobility enhancement can be attributed to the increased crystallinity and alignment in SVA-HMF film as a direct result of the controlled molecular orientation self-assembly behaviors. The carrier mobility of the SVA-HMF P(NDI2OD-T2) film with a higher degree of orientation exhibits a more pronounced enhancement. The increase in charge carrier mobility is fully consistent with AFM and 2D-GIWAXS measurements. In D-A polymers, charge carriers predominantly flow along the chain backbones and are accompanied by interchain hopping within the crystalline domains [16,21,54]. Thus, the magnetically aligned film demonstrates an enhancement in carrier mobility for two primary reasons: (I) the film possesses longer backbone chains, which promote efficient charge transport along the chain backbones; (II) the carriers are expected to encounter substantially fewer domain boundaries when moving along the alignment direction between the source and drain electrodes. For the two NDI-based polymers, magnetic energy, serving as the driving force, initiates the rotation of chain aggregates and the gradual alignment of the polymer backbone chains along the magnetic field. Meanwhile, the chain aggregates aligned in the same direction transform into more highly ordered aggregates through chain interaction. Therefore, polymers with stronger magnetic response intensities can form ordered films with a higher degree of order and larger fibril-like microstructures. Consequently, for magnetically aligned films, the higher the magnetic response characteristics, the more significant the improvement in the electrical properties of the film.

## 4. Conclusions

In this work, we have elucidated the impact of alkyl side chain length in NDI-based polymers on their pre-aggregation behavior, molecular packing, magnetic response, and subsequent performance in OFETs. The P(NDI2HD-T2) polymers with the shorter alkyl side chain length showed a higher magnetic responsiveness compared to the P(NDI2OD-T2) polymers. And the SVA-HMF P(NDI2HD-T2) film exhibited a highly aligned microstructure, demonstrating an exceptionally high value of DR up to 25.2. We have determined that reducing the alkyl side chains enhances pre-aggregation of polymer molecules, facilitating polymer alignment under a magnetic field, that is, the response of polymer molecules to the magnetic field is enhanced. The present study further enhances the theoretical understanding of polymer molecular orientation induced by magnetic fields. These property–microstructure–performance correlations established in the model system of NDI-based polymers provided valuable guidelines for selecting the optimal polymers for magnetic alignment and demonstrated the importance of the magnetic alignment method in achieving high-performance electrical devices.

## Figures and Tables

**Table 1:** Performance properties of the OFETs based on series P(NDI2HD-T2) and P(NDI2OD-T2) films.

	Deposition	μ_e_ (cm^2^/Vs)	Vth (V)	I_on_/I_off_	Note
P(NDI2HD-T2)	spin-coat	0.11 ± 0.04	12.3	10^3^~10^4^	μ_//_/μ_spin_ = 13.5
	SVA	0.15 ± 0.05	10.5	10^5^	
	SVA-HMF (//)	1.49 ± 0.31	10.0	10^5^	
	SVA-HMF (⊥)	0.078 ± 0.03	9.8	10^4^	
P(NDI2OD-T2)	spin-coat	0.085 ± 0.04	9.9	10^4^	μ_//_/μ_spin_ = 8.9
	SVA	0.13 ± 0.05	9.1	10^5^	
	SVA-HMF (//)	0.76 ± 0.23	10.2	10^5^	
	SVA-HMF (⊥)	0.007 ± 0.005	18.5	10^3^	

^a^ average values of electron carrier mobility are calculated from 16 devices.

## Data Availability

The original contributions presented in this study are included in the article and Supplementary Materials. Further inquiries can be directed to the corresponding authors.

## Supplementary Materials

The following are available online at https://www.mdpi.com/article/10.3390/polym18111328/s1. Figure S1: GPC curves of the P(NDI2HD-T2) and P(NDI2OD-T2) polymers; Figure S2: The thickness of films; Figure S3: Polarized optical microscopy images of the SVA-HMF P(NDI2HD-T2) and P(NDI2OD-T2) films; Figure S4: AFM phase images of the series films; Figure S5: Cross-section profiles of the (001) reflection; Figure S6: The optical bandgap of P(NDI2HD-T2) and P(NDI2OD-T2); Figures S7 and S8: Output curves for TG/BC OFET devices [55,56,57].
