# Supplementary material for: Impact of Alkyl Side Chain Length on Morphological Properties and Magnetic Field Response Characteristics of Naphthalenediimide-Based Conjugated Polymer
Source: Polymers (Basel). 2026 May 27;18(11):1328. doi: 10.3390/polym18111328 (PMC13259258; doi:10.3390/polym18111328)
Supplement: Supplementary file 1 [file polymers-18-01328-s001.zip › polymers-4299937-supplementary.pdf]

polymers
MDPI

Supporting Information

Impact of Alkyl Side Chain Length on Morphological Properties and Magnetic Field Response Characteristics of Naphthalenediimide-Based Conjugated Polymer

Shichao Chen [1,†], Yiqian Zhou [1,†], Zitong Zhang [1], Xiaocan Zhang [1], Di Hui [2], Yuhan Zhang [1], Yurou He [1], Kai Zhang [2], Yingzheng Ge [1], Ziyan Feng [1], Lin Hu [2], Chun Ye [2,*] and Guoxing Pan [1,*]

[1] Institutes of Physical Science and Information Technology, Anhui University, Hefei 230601, China; chenshichao2023@163.com (S.C.); zhouyiqian0728@163.com (Yiqian Zhou); ztzhang2024@163.com (Z.Z.); xczhang20000527@163.com (X.Z.); zhangyuhanahu@163.com (Yuhan Zhang); heyurouahu@163.com (Y.H.); geyz0202@163.com (Y.G.); fzy20250919@163.com (Z.F.)

[2] Anhui Province Key Laboratory of Condensed Matter Physics at Extreme Conditions, High Magnetic Field Laboratory (HMFL), Chinese Academy of Sciences, Hefei 230031, China; huidi021@163.com (D.H.); zeki@mail.ustc.edu.cn (K.Z.); hulin@hmfl.ac.cn (L.H.)

* Correspondence: cye927@hmfl.ac.cn (C.Y.); panguoxing@ahu.edu.cn (G.P.)

† These authors contributed equally to this work.

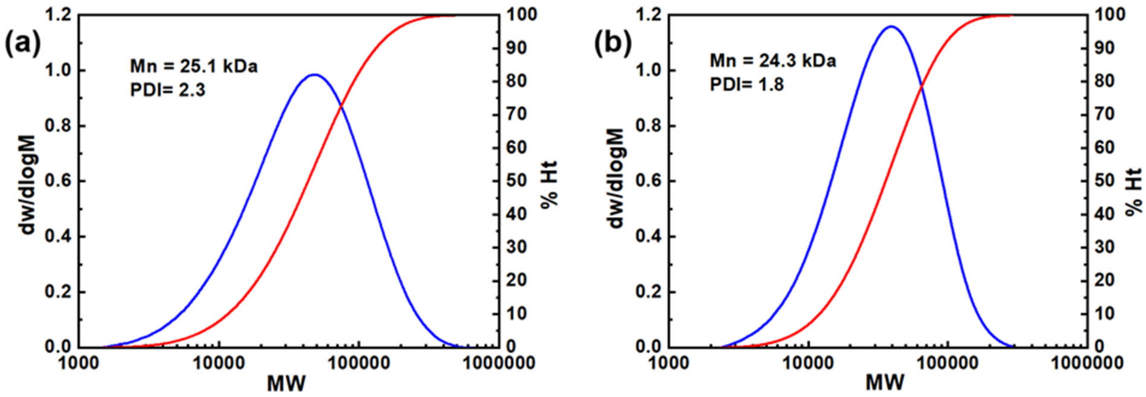

Figure S1. GPC curves of the P(NDI2HD-T2) (a) and P(NDI2OD-T2) (b), respectively.

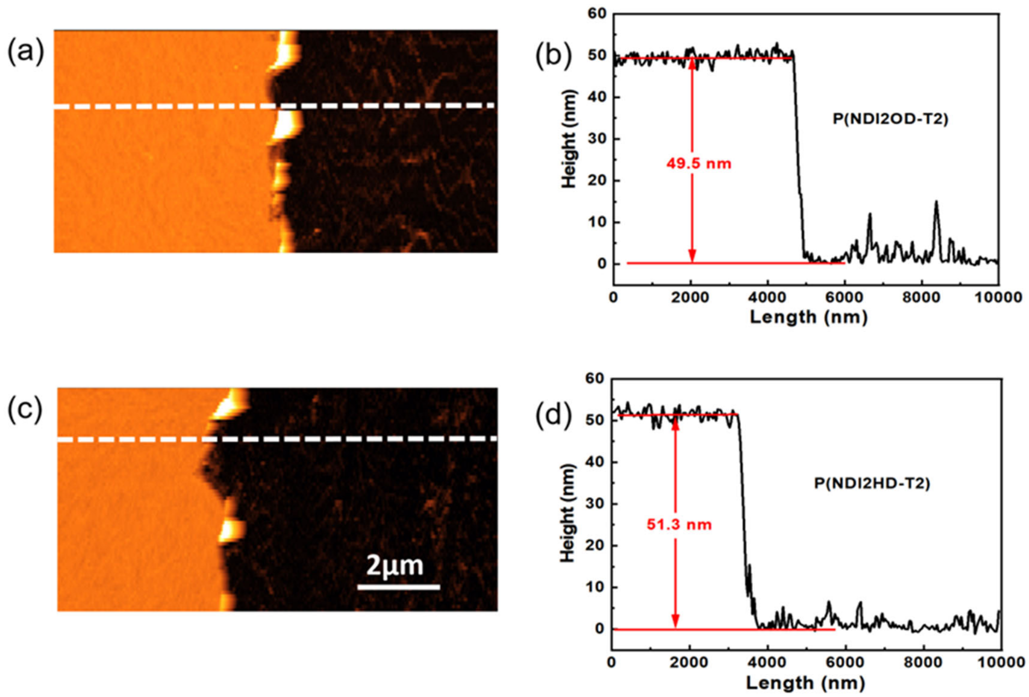

Figure S2. AFM measurement to determine film thickness of the SVA-HMF P(NDI2OD-T2) (a, b) and P(NDI2HD-T2) (c, d) films, respectively.

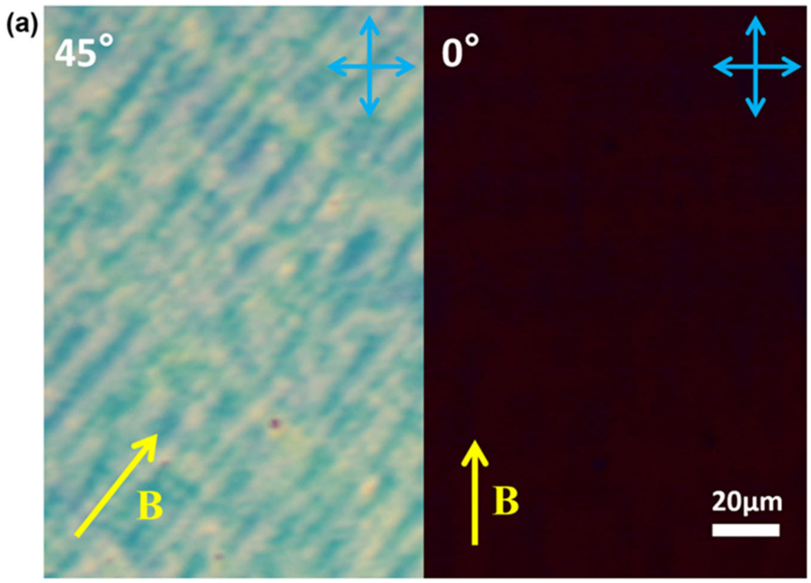

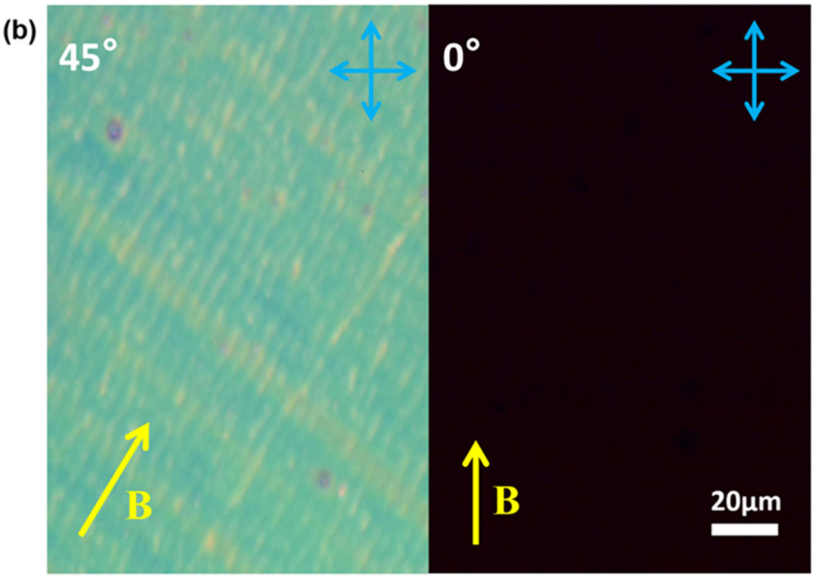

Figure S3. POM images of SVA-HMF P(NDI2OD-T2) (a) and P(NDI2HD-T2) (b) films, respectively. The yellow arrows denote the HMF direction.

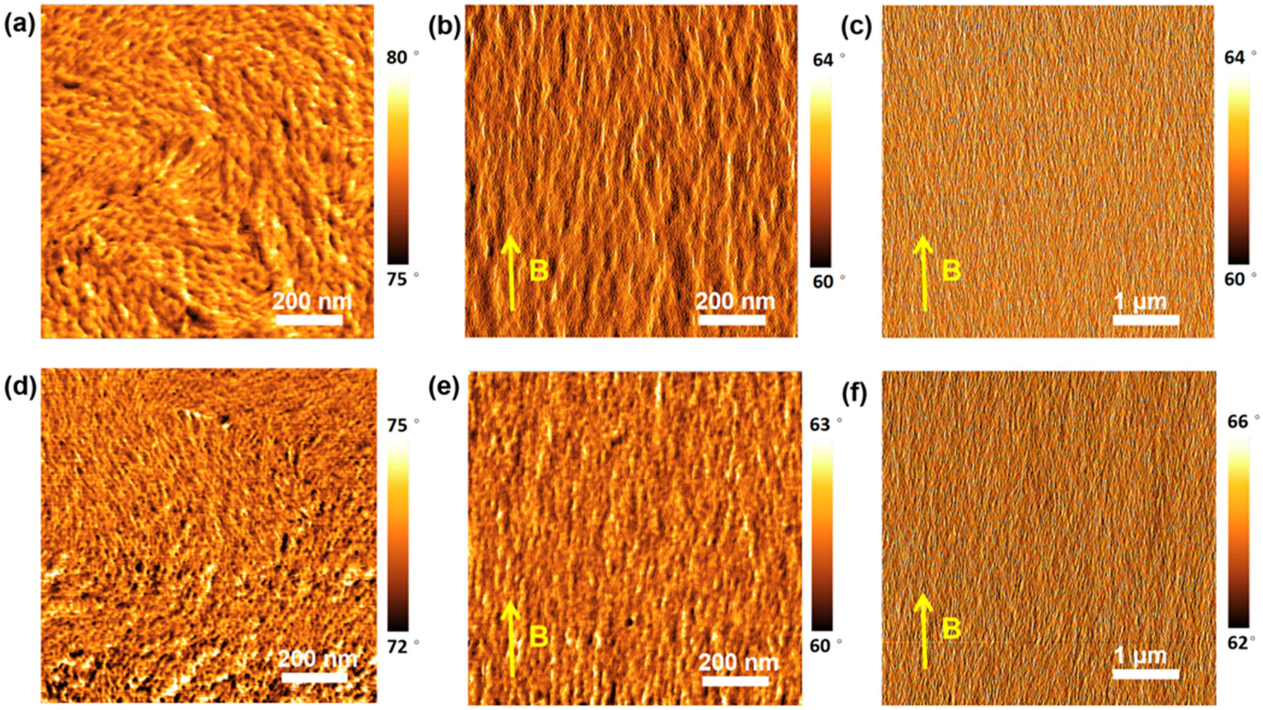

Figure S4. AFM phase images of P(NDI2HD-T2) (a, b, c) and P(NDI2OD-T2) (d, e, f) films, respectively. And the films were prepared by spin-coat (a, d) and SVA-HMF (b, c, e, f) methods, respectively. The yellow arrows denote the HMF direction.

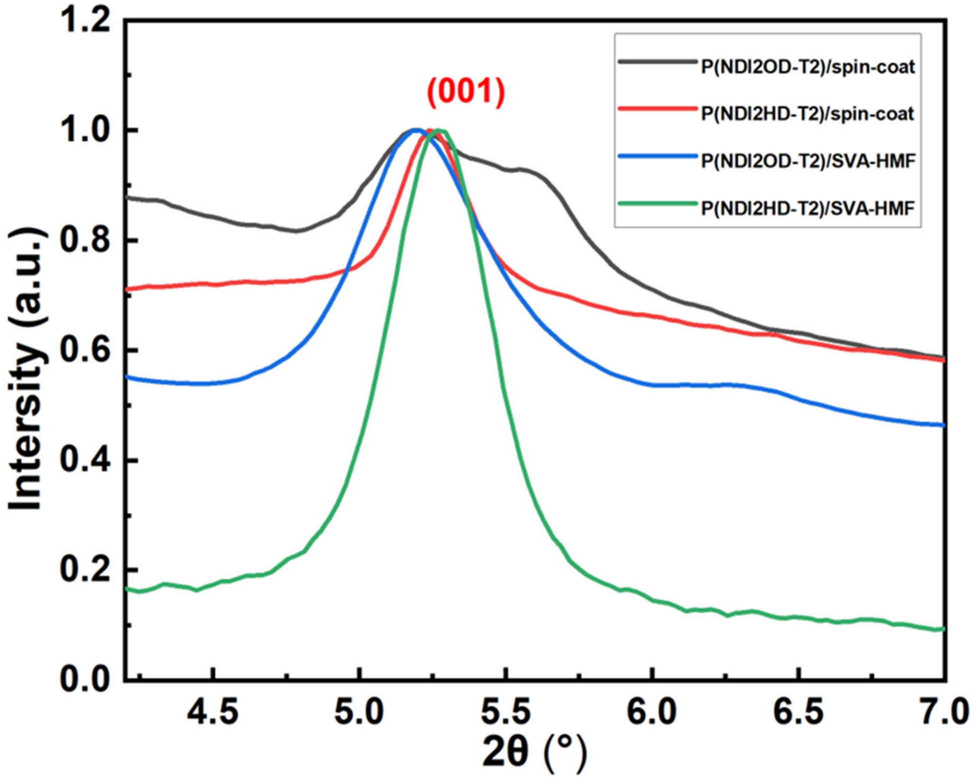

Figure S5. Cross-section profiles of the (001) reflection of P(NDI2OD-T2) and P(NDI2HD-T2) films shown in Figure 3. The crystalline correlation length (CCL) is estimated from the full width at half maximum (FWHM) of the (001) peak based on Scherrer equation [55-57] as follow:

$$D = \frac{\mathrm{k}\lambda}{\mathrm{B}\cos\theta} \quad (1)$$

where D is the crystallite size, λ is the X-ray wavelength in nanometer (0.124 nm in our experiments), K is a shape factor (typically 0.89) and B is the FWHM of diffraction peaks.

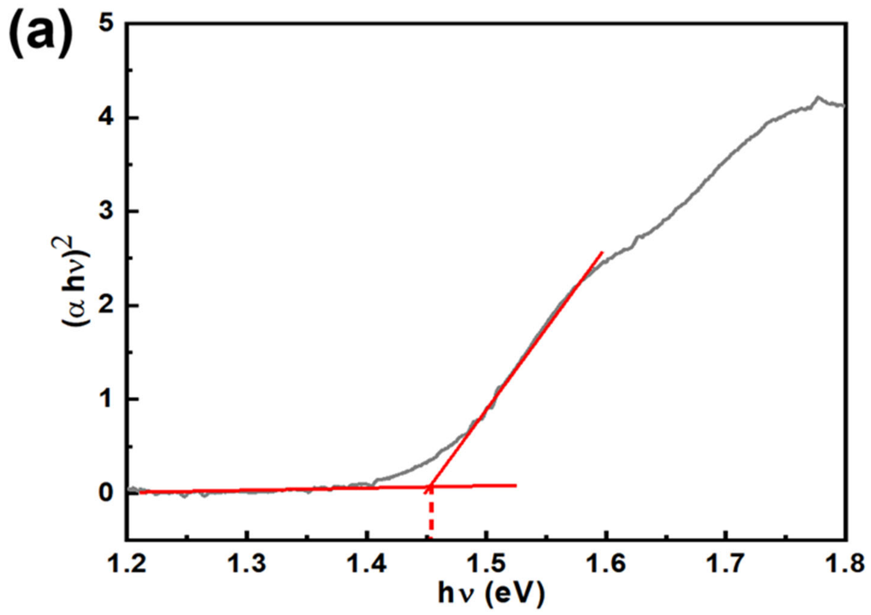

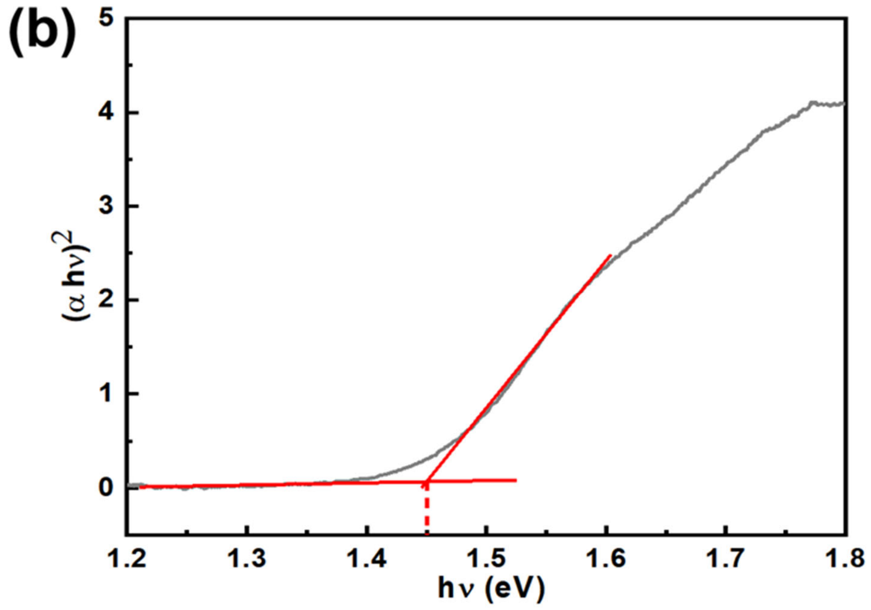

Figure S6. The tauc plot of the optical bandgap for the P(NDI2OD-T2) (a) and P(NDI2HD-T2) (b), respectively.

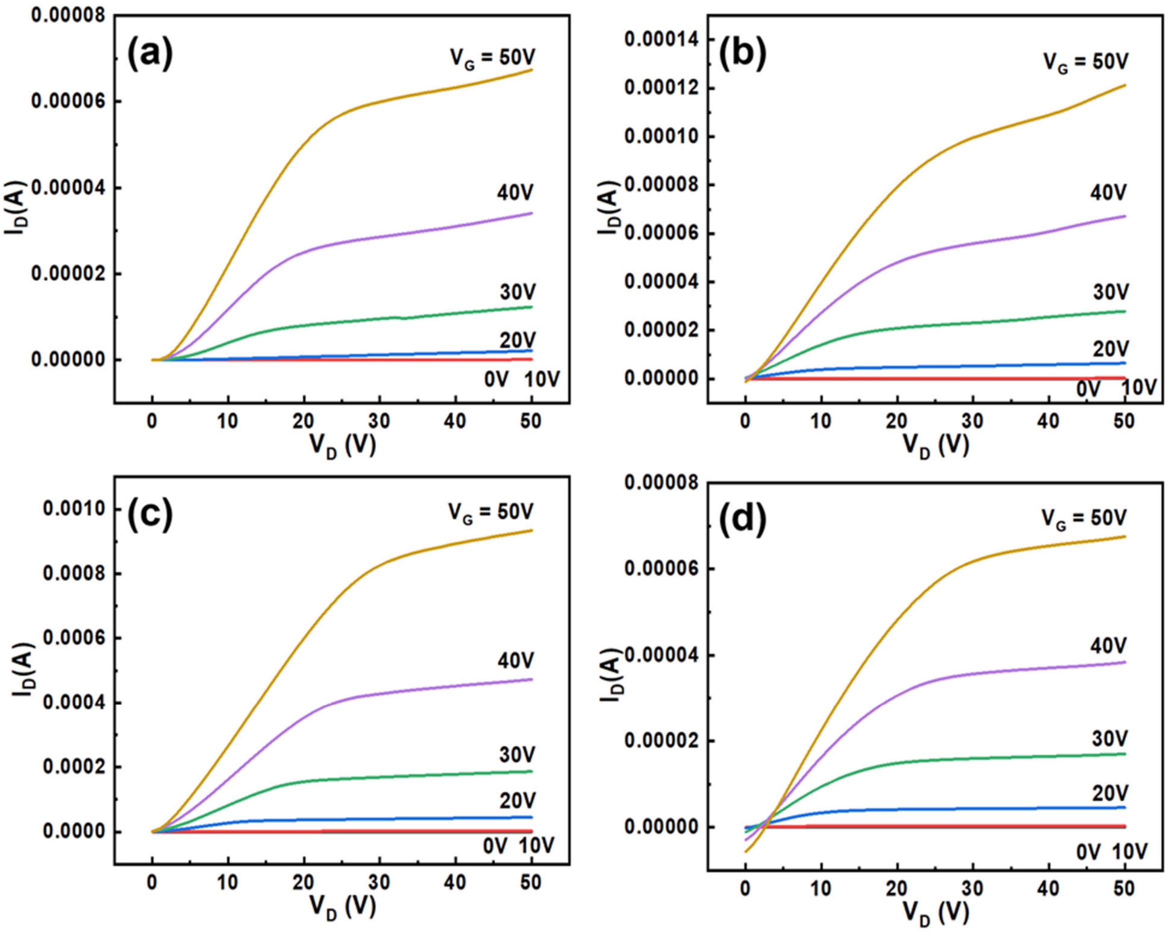

Figure S7. Typical output curves of the TG/BC OFETs (W = 2 mm and L = 5μm) of the spin-coat isotropic P(NDI2HD-T2) film (a), as well as the SVA film (b) and the magnetically aligned P(NDI2HD-T2) film. The channel current is parallel (c) and perpendicular (d) to the magnetic alignment direction, respectively.

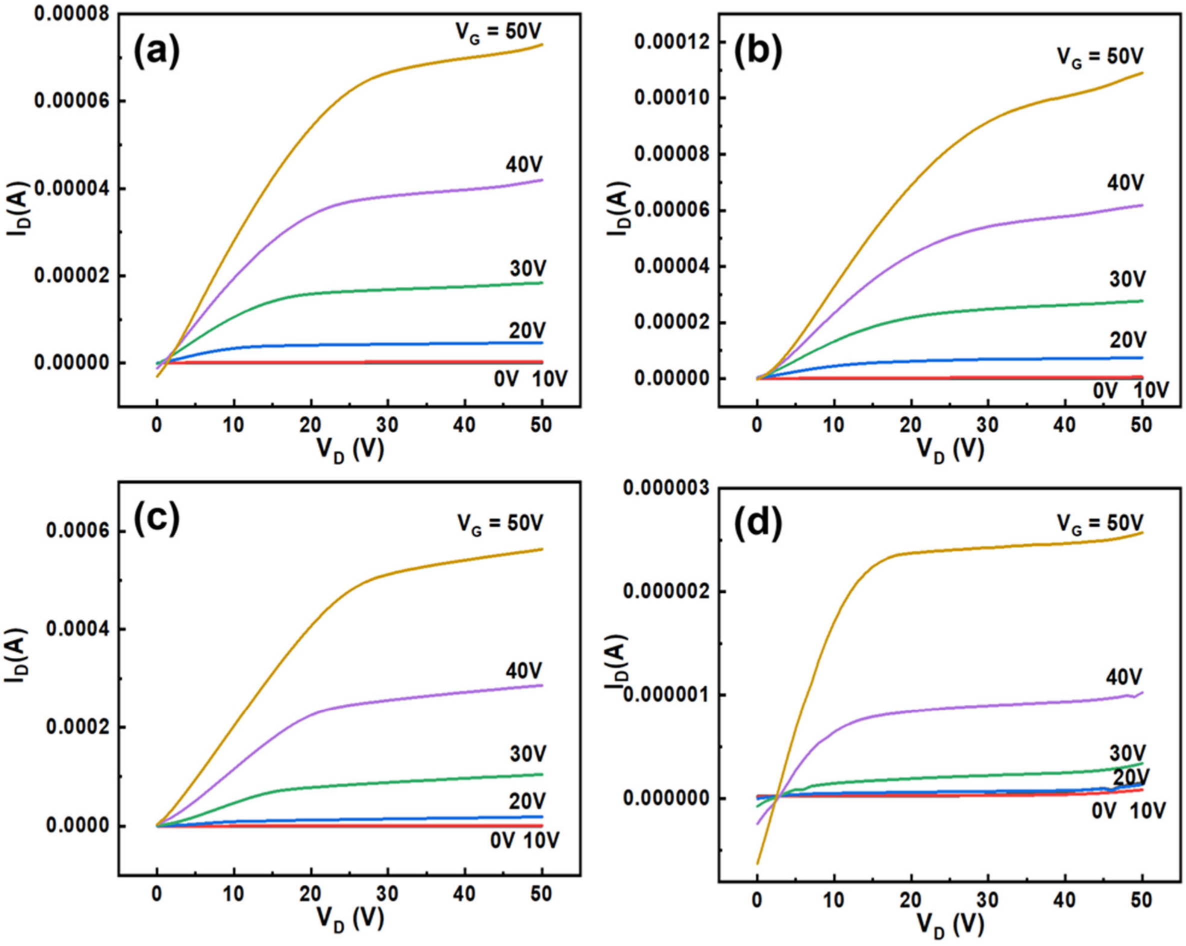

Figure S8. Typical output curves of the TG/BC OFETs (W = 2 mm and L = 5μm) of the spin-coated isotropic P(NDI2OD-T2) film (a), as well as the SVA film (b) and the magnetically aligned P(NDI2OD-T2) film. The channel current is parallel (c) and perpendicular (d) to the magnetic alignment direction, respectively.

References

55 McGehee, R.; RMcGehee, J. The Use of Standard Deviation of X-ray Diffraction Lines as a Measure of Broadening in the Seherrer Equation: A Curve Fitting Method. *J. Appl. Cryst.* **1972**, *5*, doi:10.1107/S002188987200977X.

56 Muniz, F. T. L.; Miranda, M. A. R.; Morilla dos Santos, C.; Sasaki, J. M., The Scherrer equation and the dynamical theory of X-ray diffraction. *Acta Crystallogr. A.* **2016**, *72*, doi:10.1107/S205327331600365X.

57 Gu, X.; Yan, H.; Kurosawa, T.; Schroeder, B.C.; Gu, K.L.; Zhou, Y.; To, J.W.F.; Oosterhout, S.D.; Savikhin, V.; Molina-Lopez, F.; Tassone, C.J.; Mannsfeld, S.C.B.; Wang, C.; Toney, M.F.; Bao, Z. Comparison of the Morphology Development of Polymer–Fullerene and Polymer–Polymer Solar Cells during Solution-Shearing Blade Coating. *Adv. Energy Mater.* **2016**, *6*, doi:10.1002/aenm.201601225.
